# Socioeconomic position and the effect of energy labelling on consumer behaviour: a systematic review and meta-analysis

**DOI:** 10.1186/s12966-023-01418-0

**Published:** 2023-02-06

**Authors:** Eric Robinson, Megan Polden, Tess Langfield, Katie Clarke, Lara Calvert, Zoé Colombet, Martin O’Flaherty, Lucile Marty, Katy Tapper, Andrew Jones

**Affiliations:** 1 Department of Psychology, University of Liverpool, Eleanor Rathbone Building, Bedford Street South, Liverpool, L69 7ZA UK; 2 Department of Public Health Policy and Systems, University of Liverpool, Liverpool, UK; 3 Centre Des Sciences Du Goût Et de L’Alimentation, CNRS, INRAE, Institut Agro, Université Bourgogne Franche-Comté, 21000 Dijon, France; 4 Department of Psychology, City, University of London, London, UK

**Keywords:** Energy labelling, Calorie labels, Individual differences, Obesity policy

## Abstract

**Background:**

There are well documented socioeconomic disparities in diet quality and obesity. Menu energy labelling is a public health policy designed to improve diet and reduce obesity. However, it is unclear whether the impact energy labelling has on consumer behaviour is socially equitable or differs based on socioeconomic position (SEP).

**Methods:**

Systematic review and meta-analysis of experimental (between-subjects) and pre-post implementation field studies examining the impact of menu energy labelling on energy content of food and/or drink selections in higher vs. lower SEP groups.

**Results:**

Seventeen studies were eligible for inclusion. Meta-analyses of 13 experimental studies that predominantly examined hypothetical food and drink choices showed that energy labelling tended to be associated with a small reduction in energy content of selections that did not differ based on participant SEP (X^2^(1) = 0.26, *p* = .610). Effect estimates for higher SEP SMD = 0.067 [95% CI: -0.092 to 0.226] and lower SEP SMD = 0.115 [95% CI: -0.006 to 0.237] were similar. A meta-analysis of 3 pre-post implementation studies of energy labelling in the real world showed that the effect energy labelling had on consumer behaviour did not significantly differ based on SEP (X^2^(1) = 0.22, *p* = .636). In higher SEP the effect was SMD = 0.032 [95% CI: -0.053 to 0.117] and in lower SEP the effect was SMD = -0.005 [95% CI: -0.051 to 0.041].

**Conclusions:**

Overall there was no convincing evidence that the effect energy labelling has on consumer behaviour significantly differs based on SEP. Further research examining multiple indicators of SEP and quantifying the long-term effects of energy labelling on consumer behaviour in real-world settings is now required.

**Review registration:**

Registered on PROSPERO (CRD42022312532) and OSF (https://doi.org/10.17605/OSF.IO/W7RDB).

**Supplementary Information:**

The online version contains supplementary material available at 10.1186/s12966-023-01418-0.

## Introduction

Changes to dietary patterns resulting in increases in average daily energy intake at the population level are thought to have been a major cause of the global obesity problem [1, 2]. In particular, from the 1970s onwards, diets shifted towards an increasing reliance on food prepared and consumed outside of the home [3]. Meals served in the out-of-home food (OOHF) sector (e.g. restaurants, cafes, coffee-shops and takeaway outlets) tend to be high in energy [4, 5] and regular consumption of OOHF is associated with both increased energy intake [6, 7] and higher Body Mass Index (BMI) [8, 9]. Because the OOHF sector may be contributing to population level obesity, multiple countries (e.g. England, the US and regions of Canada and Australia) have recently passed legislation that require OOHF businesses to label menus with product kilocalorie (energy) information [10, 11]. Although energy labelling has been legislated in order to help the public make better informed and healthier food choices, there is some uncertainty over the effect energy labelling has on consumer behaviour [11]. Systematic reviews conducted to date have concluded that energy labelling either results in a small reduction in energy purchased in the OOHF sector or no measurable change in energy purchased [12–15]. Consistent with this, some of the largest real-world studies have found evidence of small decreases to energy purchased in the OOHF sector after the introduction of energy labelling [16, 17], although this finding has not been observed in all studies, to date [18, 19].

Socioeconomic position (SEP) is important when considering equity of population level public health approaches to improve diet as obesity and poorer quality diet are associated with lower SEP [20, 21]. Two recent systematic reviews examined the extent to which dietary ‘nudging’ interventions benefit people from lower vs. higher SEP similarly [22, 23]. Although the two reviews suggested there may be tentative evidence that the effectiveness of some types of nudging interventions may differ by SEP, both concluded that there was a lack of available evidence and these reviews did not focus on energy labelling. Lower SEP is associated with reporting being less motivated by weight management and health when making food choices [24]. In the context of energy labelling, SEP patterning of food choice motives could therefore result in energy labelling having a larger effect on the dietary choices of people from higher vs. lower SEP and in turn widen inequalities in diet. Likewise, SEP differences in nutrition knowledge [25] or health literacy [26] could result in energy labelling impacting on dietary choices differentially based on SEP. In 2015 Sarink et al. [27] narratively reviewed a limited number of studies examining whether the effect of energy labelling on consumer behaviour differs based on SEP. There was a small amount of mixed evidence indicating that in some studies participants from lower SEP backgrounds were more likely to self-report noticing and using energy labelling information, but studies tended not to report results on the effect of labelling on actual consumer behaviour by SEP. Because of this the authors concluded that there was insufficient evidence to conclude whether the effect energy labelling has on consumer behaviour differs based on SEP [27]. Since the Sarink review was conducted, a number of studies have examined the impact OOHF energy labelling has on consumer behaviour in higher vs. lower SEP groups [28–31]. Furthermore, several other studies have examined the effect of energy labelling on consumer behaviour and measured SEP [32–41]. Data from these studies could be retrospectively used to quantify whether the effect of energy labelling on consumer behaviour differs based on SEP.

As more countries begin to consider adopting OOHF energy labelling as a public health policy to reduce obesity and improve diet, there is a pressing need to understand the potential impact such policies may have on tackling socioeconomic inequalities [11, 42]. Therefore, the aim of the present systematic review and meta-analysis is to quantify available evidence on whether the impact that menu energy labelling has on consumer behaviour differs in participants of lower vs. higher SEP.

## Method

PRISMA guidelines were followed [43]. The review was registered on PROSPERO (CRD42022312532) and a detailed protocol and analysis plan was pre-registered on the OSF (https://doi.org/10.17605/OSF.IO/W7RDB).

### Eligibility

#### Participants

Studies of human participants (adults and children) were eligible for inclusion, apart from studies that sampled only participants with a pre-defined psychiatric or physical health condition. For studies to be eligible for inclusion, at least one measure of participant SEP was required. Examples of eligible measures included education (e.g. highest qualification/level achieved), income (e.g. equivalised household income), local area deprivation (e.g. Indices of Multiple Deprivation, other geographical/post-code related indices of area deprivation). Demographic measures related to SEP, but not a recognised direct measurement of SEP (e.g. ethnicity, food insecurity) were not eligible.

#### Intervention condition

Studies were required to have an ‘experimental’ condition (including ‘natural’ and quasi experiments) or trial arm in which energy content information (i.e. number of kcal) for food and/or drink products was provided to participants at point of food/drink selection, purchase or consumption. Studies that labelled foods and/or beverages (both alcoholic and non-alcoholic) were eligible. Because energy labelling in OOHF settings tends to be provided without any additional nutritional information (e.g., salt, saturated fat) and our aim was to provide evidence that closely aligned with real-world implementation, studies in which energy content information was provided alongside additional non-energy based nutritional information were not eligible. This approach also allowed us to isolate the impact of energy labelling from other types of nutrition information. If studies included multiple intervention conditions with varying presentation of energy labelling (e.g. standard energy labelling vs. standard energy labelling + contextual information) we included the labelling condition that most directly aligned with existing energy labelling legislation (e.g. presentation of kcal next to menu items, contextual information/statement on the menu explaining kcal requirements for the average man/woman).

#### Comparator condition

Studies were required to have a control condition or trial arm in which energy content information was not provided.

#### Outcome

Because the primary purpose of energy labelling is to promote the selection of lower energy food and/or drink products, eligible outcomes were total energy (kcal) consumed, purchased, or selected/chosen. Eligible outcomes included real-world behavioural outcomes (e.g. energy content of purchased meal in a restaurant), as well as hypothetical measures (e.g. choices from mock restaurant menus). If a study only had an outcome measure that was not directly comparable with the above, but would likely result in fewer kcal being selected (e.g. whether energy labelling increases % of sample choosing a lower energy food item) they were also eligible. Outcomes not related to selection, choice, purchase, or consumption (e.g. self-reported use of labelling) were not eligible. Each study contributed one comparison to the meta-analysis and when selecting outcome variables we favoured (in order of preference) total energy consumed, purchased, selected/chosen.

#### Setting

Studies conducted in real-world settings (e.g. restaurants or cafes), laboratory settings and online (e.g. hypothetical menu or portion size selections as part of an online experiment) were eligible for inclusion.

#### Study designs

Eligible study designs included trials and experiments in which participants were allocated to receive the intervention or control condition. Studies using pre-post designs in which outcome were examined prior to and after implementation of energy labelling were eligible. If outcome data were available at multiple post-implementation time points, we extracted data from the longest follow-up. Studies that examined outcomes in different locations that do vs. do not have energy labelling were ineligible.

### Search strategy

There have been a number of systematic reviews and meta-analyses examining the effects of energy content labelling on consumer behaviour. In scoping searches, we identified five recent systematic reviews published in the last five years that comprehensively searched for all relevant studies of energy labelling up to and including 2020 [12–15, 44]. In addition, the Sarink et al. (2016) systematic review identified relevant studies on energy content labelling and SEP published prior to 2016. To avoid duplication of effort we first accessed the above six systematic reviews and assessed all included (and excluded) studies in each review for inclusion in the present research. We also conducted new electronic database searches for the period of 2015–2022 to identify any recent eligible energy content labelling studies that may have not been covered by the above six reviews. We searched PubMed, Scopus and PsycInfo from 01/01/2015 to 24/02/2022. See online supplementary materials for search terms (examples include ‘energy’ and ‘label* and ‘socioeco*’). We also searched the OSF (including PsychArxiv and Nutrixiv), Medrxiv and SSRN pre-print archives for unpublished studies. For the electronic database searches two researchers independently completed title and abstract screening and full-text screening. One researcher identified potentially eligible articles from the existing systematic reviews and pre-print archives. A second researcher independently checked eligibility. Any instances of disagreement were resolved through discussion.

### Availability of data

We operationalised high vs. low SEP on a study-by-study basis in line with the approach used in each individual study (e.g. a study defined high vs. low SEP as having degree level education vs. a lower qualification). If studies reported measuring an indicator of SEP but did not report results/outcome data in higher vs. lower SEP participants separately then we contacted authors to request this information. In such instances we requested high vs. low SEP data based on the most commonly used indicator of SEP in other eligible studies (highest education level achieved) if more than one SEP indicator was available.

### Data extraction

Two authors independently extracted information from all articles. In addition to bibliographic information, we extracted the following information: country study was conducted in, setting (e.g. real-world, laboratory, online), participant sample and characteristics (e.g. gender), primary SEP measure, number of participants (if studies reported outcome data for more than 2 SEP groups (e.g. income quintiles) we extracted data for each, how energy labelling was delivered and presented (e.g. whether kcal and/or kJ were presented and whether any contextual information was provided to aid interpretation), study design (experiment vs. pre-post), outcome measure category (e.g. real-world, hypothetical), outcome type (e.g. total energy selected) and time of measurement relevant to implementation of energy labelling. Outcomes were routinely measured during intervention exposure (i.e. food choices made in presence of labelling) so we favoured extraction of outcome data measured at exposure (e.g. as opposed to later in the day when labelling was absent).

### Risk of bias

Risk of bias was assessed using a checklist adapted from generic study quality assessment tools [45–47], as items on any one existing risk of bias tool (e.g. Cochrane, ROBINS-I, Newcastle–Ottawa) were not comprehensive enough to cover all important methodological considerations for the types of studies included in the present review (e.g. checklists do not include items relating to demand characteristics). The checklist varied for experimental vs. pre-post studies. See online supplementary materials for checklists in full. Items related to whether studies used random allocation, had appropriate (as opposed to very small) sample sizes, addressed demand characteristics, used blinding, relied on objective measurement (as opposed to dietary recall), had negligible missing data, assessed/addressed SEP groups differing on demographics other than SEP, whether the same outlets were sampled pre-post labelling implementation and whether any additional changes to the study setting coincided with the implementation of energy labelling.

### Planned analyses

To avoid duplicity of data from studies we favoured inclusion of the two most extreme SEP groups (high vs. low) from studies in the primary meta-analysis. Due to differences in study design, we meta-analysed experimental studies and pre-post studies separately.

#### Experimental studies

Generic variance inverse meta-analysis (random effects due to expected heterogeneity) was used with SEP as a sub-group factor. The outcome in the primary meta-analysis was standardised mean difference (SMD). SMDs = 0.2, 0.5, and 0.8 are considered statistically ‘small’, ‘medium’ and ‘large’ effect sizes, respectively. Positive SMDs are indicative of greater energy selected/consumed in the no label condition vs. label condition (SMD = (Mean No label – Mean Label) / Pooled Standard Deviation for both experimental and pre-post study types). In the primary model we combined different study outcomes (e.g. energy selected, energy consumed) and SEP indicators. However, we planned to repeat the primary model when limited to individual outcome types and SEP indicator providing there were a minimum of five studies for inclusion. We also planned to report a meta-analysis in which the outcome was kcal selected (as opposed to SMD) in order to aid interpretation.

We planned to identify outliers using a standard boxplot approach and report results of meta-analyses with the outliers removed (if results change). We also computed DFBETAS values for each effect size. DFBETAS values > 1 (indicative of a > 1 change in the standard deviation of the estimated co-efficient after removal of the study) were considered influential [48]. To increase sensitivity, we also conducted leave-one-out analyses by removing each study (k) from the analyses and refitting the model. We examined evidence for publication bias in the primary analyses by examining asymmetry of the effect sizes (visual inspection of funnel plot), conducting an Egger’s test of asymmetry [49] and a Trim and Fill procedure [50]. For the Egger’s test [49], an intercept significantly different from 0 at p > 0.10 is indicative of bias. To address risk of bias, in one analysis we limited a meta-analysis to studies with no risk of bias for ≥ 75% of the individual bias indicators and in the other analysis we limited meta-analysis to studies that randomized participants to conditions and addressed demand characteristics.

#### Pre-post studies

We meta-analysed studies using the same approach as for experimental studies (SMD as the effect size). In instances where data was missing, we imputed values based on studies in which data was available (see Results section for more detail). Due to the small number of pre-post studies included in the meta-analysis, we were not able to quantitatively examine influential cases, publication bias or risk of bias. Data and analysis scripts can be found on the OSF.

## Results

### Study selection

**Fig. 1:**
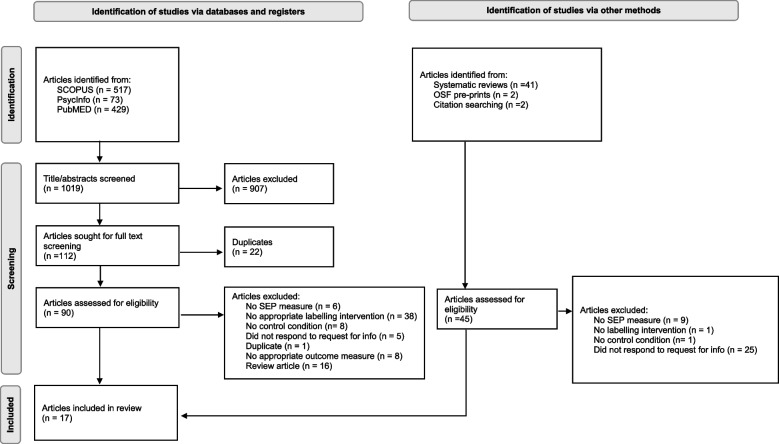
Study selection process for inclusion in systematic review

A total of 17 eligible studies were included. See Fig. 1 for study selection procedure. Thirteen of the studies were experimental and four of the studies had pre-post designs in which outcomes were measured pre and post implementation of energy labelling in real-world settings. See Table 1 for individual study information.

**Table 1:** Study characteristics

**Study**	**Country**	**Population and sample size** *N* = Total sample (*N* = sample of included conditions)	**Study setting**	**Study design**	**Contextual information provided**	**Outcome measure (kcal or kJ)**	**SEP measure used**	**SEP cut off**
Carbonneau et al. (2015) [37]	Canada	*N* = 160 (*N* = 72) (No Label condition: Mean age = 42.6yrs, Label condition: Mean age = 37.7yrs)	Field setting (real-world)	Randomised control trial (BS)	Y	Mean kcal consumed	Highest educational qualification	High = Above A-Level Low = A-Level or below
Krieger et al. (2013) [51]	US	*N* = 4087 (*N* = 4087) (Age: < 40yrs *N* = 2,189, $\ge$ 40yrs *N* = 1,872)	Field setting (real-world)	Pre-post intervention design (BS)	Not reported	Mean kcal selected	Location of chain (census tracts)	High = High-income area Low = Low-income area
Dodds et al. (2014) [52]	Australia	*N* = 329 (*N* = 217) (No label condition: Mean age = 40.2yrs, Label condition: Mean age = 38.9yrs)	Field setting (hypothetical)	Randomised control trial (BS)	Y	Mean kJ selected	Highest educational qualification	High = Degree or above Low = Below degree
Antonelli & Viera (2015) [53]	US	*N* = 823 (N = 405) (No label condition: Mean age = 38yrs, Label condition: Mean age = 38yrs)	Online (hypothetical)	Randomised control trial (BS)	N	Mean kcal selected	Highest educational qualification	High = Degree or above Low = Below degree
Marty et al. (2020) [29]	UK	*N* = 1,743 (*N* = 893) (Study1: Mean age = 35.5yrs) (Study2: Mean age = 36.1yrs)	Online (hypothetical)	Randomised control trial (BS)	Y	Mean kcal selected	Highest educational qualification	High = Above A-level Low = A-level or below
Marty et al. (2021a) [28]	UK	*N* = 1667 (*N* = 842) Mean age = 36.9yrs)	Online (hypothetical)	Randomised control trial (BS)	Y	Mean kcal selected	Highest educational qualification	High = Above A-level Low = A-level or below
Marty et al. (2021b) [30]	US	*N* = 2091 (*N* = 1051) (Study1: Mean age = 35.3yrs) (Study2: Mean age = 44.9yrs)	Online (hypothetical)	Randomised control trial (BS)	Y	Mean kcal selected	Highest educational qualification	High = Above high school Low = High school or below
Maynard et al. (2018) [54]	UK	*N* = 264 (*N* = 260) (No label condition: Mean age = 23yrs, label condition: Mean age = 22.8yrs,	Laboratory (real-world)	Randomised control trial (BS)	N	Mean percentage consumed	Highest educational qualification	High = Above A-level Low = A-level or below
Al-Otaibi (2021) [55]	Saudi Arabia	*N* = 333 (No label condition: Mean age = 20.5yrs, Label condition: Mean age = 20.3yrs)	Field (hypothetical)	Control trial (BS)	N	Mean kcal selected	Household income	High = Higher monthly household income (> 5000Sr) Low = Lower monthly household income (< 5000Sr)
Morley (2013) [56]	Australia	*N* = 1294 (*N* = 515) (No Label condition: 18-29yrs 25.1%, 30-39yrs 37.1%, 40-49yrs 37.8%; Label condition: 18-29yrs 22.7%, 30-39yrs 35.5%, 40-49yrs 41.8%)	Online (hypothetical)	Randomised control trial (BS)	Y	Mean kJ selected	Highest educational qualification	High = Tertiary education or above Low = Below tertiary education
Walker et al. (2019) [57]	New Zealand	*N* = 615 (*N* = 524) (Mean age = 41.2yrs)	Online (hypothetical)	Randomised control trial (BS)	N	Mean number selected	Household income	High = Higher household income (> 60,000NZD) Low = Lower household income (≤ 60,000NZD)
Dumanovsky et al. (2011) [58]	US	*N* = 15,789 (*N* = 9787) (Age: $\le$ 34yrs *N* = 4083, > 34yrs *N* = 3965) (Pre condition data not reported)	Field setting (real-world)	Pre-post intervention design (BS)	Not reported	Mean kcal selected	Location of chain	High = Higher income area (< 25% households below poverty level) Low = Lower income area (> 45% below poverty level)
Elbel et al. (2013) [59]	US	*N* = 1169 (1169) (Age: < 40 yrs *N* = 585, ≥ 40 yrs N = 584)	Field setting (real-world)	Pre-post intervention design (BS)	Not reported	Mean kcal selected	Highest educational qualification	High = Above high school Low = High school or below
Petimar et al. (2019) [31]	US	*N* = 49,062,440 (No. of transactions) (Age not reported)	Field setting (real-world)	Pre-post intervention design (BS)	Y	Mean kcal selected	Highest educational qualification	High = Higher household income (> 50 329USD) Low = Lower household income (< 50 329USD)
Masic et al. (2017) [32]	UK	*N* = 458 (*N* = 192) (No label condition: Mean age = 30.8 yrs, Label condition: Mean age = 28.45 yrs)	Online (hypothetical)	Randomised control trial (BS)	N	Mean kcal selected	Location of chain	High = Higher income area (5–4 IMD score) Low = Lower income area (1–3 IMD score)
Van Epps et al. (2021) [60]	US	*N* = 2820 (*N* = 1881) (Mean age = 36.6 yrs)	Online (hypothetical)	Randomised control trial (BS)	N	Mean kcal selected	Household income	High = Higher household income (≥ 62,500$US) Low = Lower household income (< 37,500$US)
Robertson & Lunn (2020) [34]	Ireland	*N* = 142 (*N* = 138) (No label condition: Mean age = 40.49 yrs, Label condition: Mean age = 39.28 yrs)	Laboratory setting (non-hypothetical)	Randomised control trial (BS)	Y	Mean kcal selected	Highest educational qualification	High = Degree or above Low = Below degree

Contextual information relates to whether or not information aiding interpretation (e.g. ‘the average woman needs 2000 kcal per day’) was provided alongside energy labelling

*BS* between subjects, *IMD* Indices of multiple deprivation, *Sr* Saudi riyal, *UK* United Kingdom, *US* United States, *USD* United States dollar, *Y* Yes

### Experimental studies characteristics

Of the 13 experimental studies, most were conducted with UK (*n* = 4), North American (*n* = 3) or Australasian (*n* = 3) samples. Other studies (*n* = 1) were conducted in Canada (*n* = 1), Saudi Arabia (*n* = 1), and Ireland (*n* = 1). All studies sampled men and women (except for one laboratory study that sampled women only [37]). The most common SEP measure was highest education level (9/13), followed by household income (3/13) and one study used household postcode (1/13). High SEP education level was classed as qualifications above A-levels in 4/9 studies, degree or above in 3/9, above high school in 1/9 and tertiary education or above in 1/9 studies. For household income, one study classed high SEP as a monthly household income of > 5000 Saudi riyal, one study classed high SEP as an annual household income > 60,000 NZD and one study classed high SEP as an annual income of > 62,500 USD. One study using household postcode classed high SEP as an Index of Multiple Deprivation (IMD) score of 4 or 5. Eight of the 13 studies were conducted online using a hypothetical outcome measure, 2/13 were conducted in a real-world setting using a hypothetical outcome measure, 3/13 examined actual food/drink selection (of these 2 were in laboratory settings and 1 were in real-world settings). When assessing risk of bias, 12/13 studies used random allocation, 3/13 had very small sample sizes (n < 20 in one or more SEP groups), 6/13 studies reported addressing demand characteristics, in 7/13 studies the assessors were blinded and unaware of the intervention condition assigned to the participant, all 13 studies used objective measures to record purchases such as receipts, none of the 13 studies reported high levels of missing data (> 10% missing data) and for 12/13 studies the SEP groups did not differ and were comparable in terms of other demographics. See online supplementary materials for study level risk of bias ratings.

### Pre-post studies characteristics

Of the 4 pre-post studies, all were conducted in the US. Two of the four studies used chain location to operationalise SEP, one study used education level (high SEP = above high school education) and one study used household income (high SEP =  > 50,329 USD). Of the 2 studies that used chain location to indicate SEP, one of the studies classed high SEP as < 25% households in that area below poverty level, and one classed high SEP as < 35% of residents below 200% of the federal poverty level. All 4 of the studies were conducted in real-world settings and examined actual food/drink selections. For risk of bias, none of the studies included had very small sample sizes (n > 20 in all SEP groups), none of the studies had the assessors blinded or unaware of the intervention condition, all 4 of the studies used objective measures to record purchases such as receipts, none of the 4 studies reported high levels of missing data, 3/4 studies reported group differences in terms of demographics, 1/4 analysed weekly purchases and therefore did not report person level data, 3/4 of the studies participants were aware that they were involved in the study when making their purchases and for 3/4 studies the same outlets were included for the pre/post assessments. See online supplementary materials for study level risk of bias ratings.

### Meta-analysis: experimental studies

#### Primary analyses

**Fig. 2:**
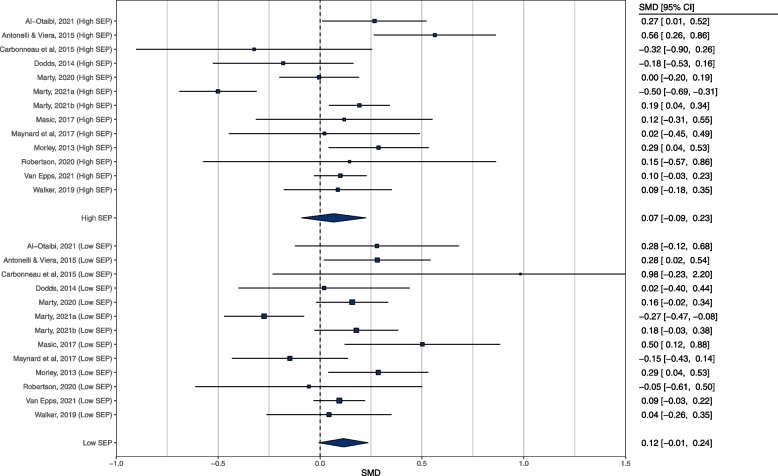
Primary meta-analysis for experimental studies

**Fig. 3:**
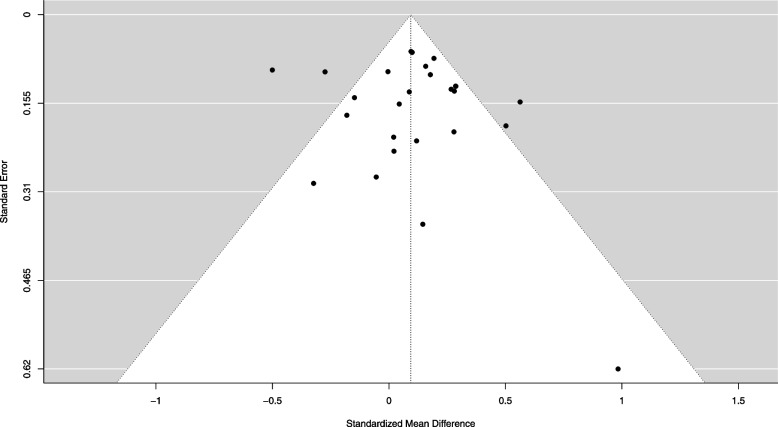
Funnel plot for experimental studies in primary meta-analysis

A total of 26 effects (13 studies) were meta-analysed in the primary model (16 effect sizes were generated from the outcome kcal selected, 4 from kJ consumed, 2 from kcal consumed, 2 from percent of total meal consumed, 2 from number of beverages consumed). In the overall meta-analysis of included eligible studies there was no significant change in consumer behaviour as a result of the presence of kcal labelling (*n* = 26: SMD = 0.094 [95% CI: -0.005 to 0.193], Z = 1.85, *p* = 0.064, I^2^ = 73.1%). There was no significant moderation by SEP (X^2^(1) = 0.26, *p* = 0.610). Higher SEP SMD = 0.067 [95% CI: -0.092 to 0.226], while in lower SEP the effect was SMD = 0.115 [95% CI: -0.006 to 0.237]. See Fig. 2. Egger’s test for funnel plot asymmetry was not significant (Z = 0.752, *p* = 0.451). See Fig. 3 for funnel plot. Trim and Fill analysis estimated the inclusion of 5 studies to obtain funnel plot symmetry and their inclusion changed the pooled effect size to SMD = 0.028 [-0.074 to 0.131], indicating a small degree of potential publication bias. There were 2 outliers (SMD = 0.98 and SMD = -0.50) as identified by a boxplot. Removal of these effects changed the overall effect of kcal labelling and resulted in it being statistically significant (SMD = 0.121 [95% CI: 0.040 to 0.201], Z = 2.95, *p* = 0.003, I^2^ = 55.9%), with energy labelling associated with reduced consumption. Critically, the moderation effect of SEP remained non-significant (X^2^(1) = 0.137, *p* = 0.711). There were no individual studies with DFBETAs > 1. Leave one out analysis demonstrated that removal of the largest effect size (SMD -0.50) also made the overall effect of kcal labelling significant (SMD = 0.124 [95% CI: 0.044 to 0.204], Z = 3.04, *p* = 0.002, I^2^ = 55.1%), and when this effect was removed the moderation by SEP remained non-significant (X^2^(1) = 0.079, *p* = 0.779).

### Secondary analyses

Analyses below include the outlying effect sizes (although if there was a change in the interpretation as a result of the exclusion this is reported).

#### Energy selected

**Fig. 4:**
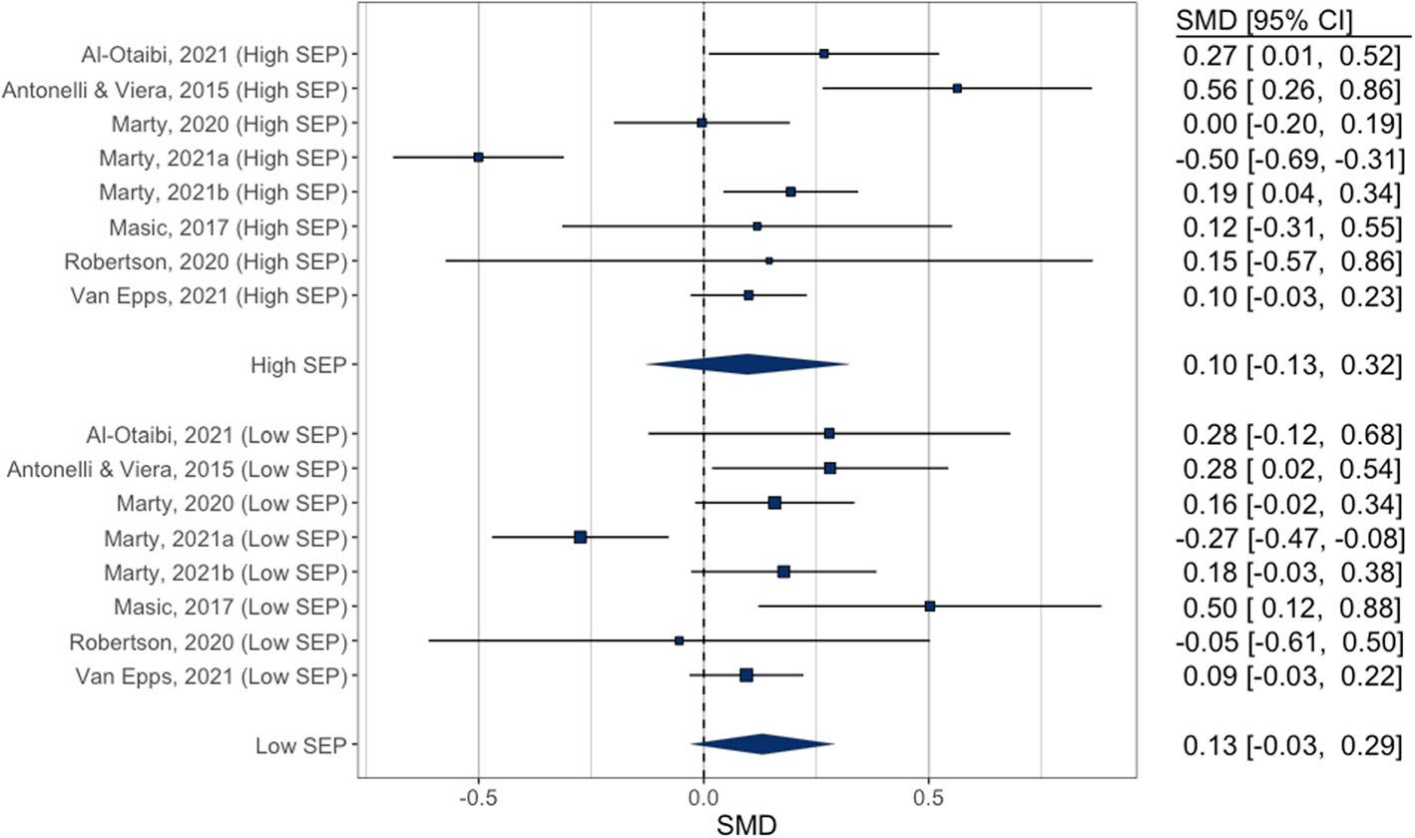
Meta-analysis limited to studies examining energy selected only

Examination of studies which used the outcome kcal selected was available (*n* = 16 effects) demonstrated no significant main effect, as shown in Fig. 4; SMD = 0.114 ([95% CI: -0.021 to 0.250], Z = 1.66, *p* = 0.097, I^2^ = 82.3%), which was not moderated by SEP (X^2^(1) = 0.090, *p* = 0.764). In higher SEP the effect was SMD = 0.098 [95% CI: -0.128 to 0.323] and in lower SEP the effect was SMD = 0.130 [95% CI: -0.029 to 0.290]. There was a significant main effect of kcal selected when outlying effect sizes were removed (*n *= 15: SMD = 0.155 [95% CI: 0.051 to 0.258]), however the moderation by SEP level was non-significant (X^2^(1) = 0.324, *p* = 0.569). When kcal selected was the outcome variable (as opposed to SMD) there was also a significant effect of energy labelling reducing kcals selected (39.79 kcal ([95% CI: -4.87 to -74.72], Z = 2.23, *p* = 0.025), but no moderation by SEP (see online supplementary materials).

#### Education level

Limiting analyses to studies that quantified SEP using highest education qualification (*n* = 18 effects), there was no effect of kcal labelling (SMD = 0.057 [95% CI: -0.081 to 0.194], Z = 0.81, *p* = 0.417, I^2^ = 77.2%) and no moderation by SEP level (X^2^(1) = 0.150, *p* = 0.698). In higher SEP the effect was SMD = 0.028 [95% CI: -0.202 to 0.259] and in lower SEP the effect was SMD = 0.081 [95% CI: -0.079 to 0.240].

#### Risk of bias analyses

Results were consistent (no moderation by SEP) across risk of bias analyses (see online supplementary materials).

### Meta-analysis: pre-post studies

**Fig. 5:**
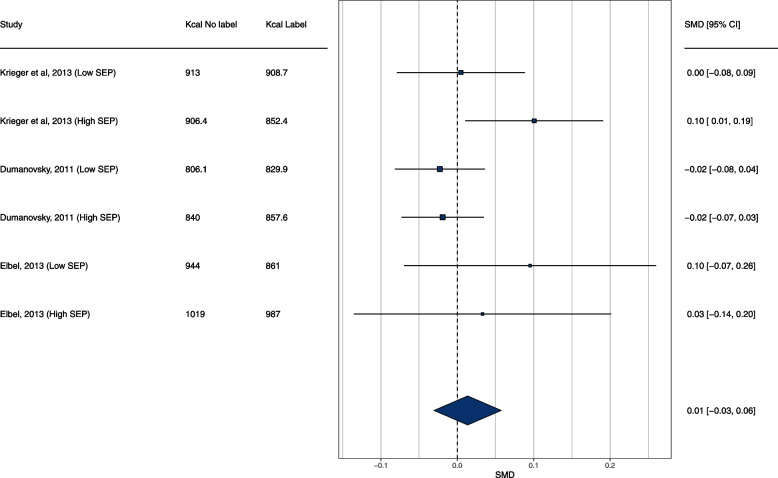
Pre-post studies meta-analysis

A total of 3 studies were meta-analysed. One study (2 effects) did not provide SDs so these were conservatively imputed as the proportion (%) of the SD/MEAN from the available studies in each condition (~ 104% of the mean in the no label condition and 92% of the mean in the label condition). In the overall meta-analysis of included eligible studies there was no significant effect of kcal labelling (SMD = 0.014 [95% CI: -0.031 to 0.057], Z = 0.60, *p* = 0.546, I^2^ = 36.5%), whereby energy purchased was non-significantly lower post as opposed to pre-implementation of energy labelling. Moderation by SEP was not significant (X^2^(1) = 0.22, *p* = 0.636). In higher SEP the effect was SMD = 0.032 [95% CI: -0.053 to 0.117] and in lower SEP the effect was SMD = -0.005 [95% CI: -0.051 to 0.041]. See Fig. 5. A further fourth study [31] was not included in the meta-analysis as it used an interrupted time series analysis to examine trend change in energy purchased pre vs. across year after implementation of energy labelling in a US restaurant franchise and was therefore not directly comparable. The study reported no SEP differences in energy purchased per transaction immediately after energy labelling was implemented, but reported that a post-implementation trend of increasing energy per purchase was stronger in lower (median) income census tracts (level change in kcal per transaction/week 0.94 [95% CIs: 0.67 to 1.21] than in higher income census tracts 0.50 [95% CIs: 0.19 to 0.81], although a formal statistical comparison of lower vs. higher income tracts was not reported (i.e. whether the post-implementation trend was statistically moderated by income tract).

## Discussion

We systematically reviewed and meta-analysed the effect of energy labelling on consumer behaviour in participants of lower vs. higher SEP in a limited number of studies which examined energy labelling and also included measurement of SEP. Among the 17 studies included in the present review, 13 used experimental designs (between-subjects) to compare energy selected in the presence vs. absence of energy labelling and the majority of these studies examined hypothetical food choices. SEP in these studies tended to be defined through participant-level education. The other four studies included were conducted in real-world settings and compared energy purchased in OOHF settings pre vs. post implementation of energy labelling. These studies defined SEP through measures of local area deprivation, comparing energy purchased in OOHF outlets in less vs. more affluent areas. In meta-analyses of both study types, there was no evidence that the effects energy labelling had on consumer behaviour significantly differed based on SEP.

The meta-analyses in this review were limited to studies which included measurement of SEP yet overall results tended to be consistent with evidence from comprehensive reviews indicating that energy labelling is associated with a small reduction in the energy content of food selections [11]. This observation provides more confidence in the present results which indicated no evidence of moderation by SEP. However, it is important to note that because we only included energy labelling studies that measured and allowed for stratification of results by SEP, the present results should not be interpreted as systematic evidence on the overall effect energy labelling has on consumer behaviour. The key finding of the present research is that we found no evidence of moderation by SEP and this finding appears to be inconsistent with some observational evidence that participants from lower SEP are less likely to report noticing and reporting using energy labelling [27]. Yet, it is important to note that SEP patterning of self-reported energy labelling noticing and usage has not been consistently observed in previous studies and such reports are also likely to be influenced by reporting bias [27]. It has been hypothesised that information based health policies, such as energy labelling, may benefit higher SEP groups more so than lower SEP groups and in doing so widen inequalities in diet and health [42]. Lower SEP has been shown to be associated with being less likely to be motivated by health and weight management when making food choices and based on this one prediction is that energy labelling may have less impact on food choice among lower SEP groups [24]. However, there are plausible alternative reasons why energy labelling could impact food choice among some subgroups of people from lower SEP backgrounds more so than higher SEP groups. For example, because lower SEP is associated with possessing less nutritional knowledge (e.g. less aware of food energy content) [61], the presence of energy labelling may be more informative and impactful. Further research identifying under which conditions or contexts energy labelling impacts consumer behaviour among higher and lower SEP groups will be informative. Although we found no evidence that energy labelling affects consumer behaviour differently based on SEP, there are other pathways through which energy labelling policies could affect socioeconomic inequalities in diet. Energy labelling policies may result in reformulation of food and drink energy content [62] and dependent on which types of outlets reformulate energy content and how frequently food from such outlets are consumed, impacts on total energy consumed from OOHF sources may therefore vary across population sub-groups [63]. Nonetheless, the lack of evidence for energy labelling having a differential effect based on SEP suggests that energy labelling is unlikely to further widen socioeconomic inequalities in diet quality and obesity.

There are limitations to the present research. There were only a relatively small number of eligible studies. In particular, the meta-analysis of pre-post implementation studies included only 3 studies and all were conducted in the US. Of the 13 experimental studies, most relied on hypothetical food choice as the outcome variable and findings may differ under real world conditions. Due to available data we were only able to examine a limited range of SEP indicators and in experimental studies most defined SEP through education level. It may be the case that SEP indicators relating to financial resources (e.g. household income, total wealth) result in a greater need to prioritise other food choice motives (such as time or price) which may result in energy labelling being less impactful on food choice. Therefore, findings may differ when alternative measures of SEP are used and this is a limitation of the present review. Studies also tended to divide higher vs. lower SEP into two broad groups (e.g. university educated vs. no university education). SEP differences may only occur at more extreme dichotomies (e.g. lowest income quintile, no formal education qualifications vs. high income, highly educated). Future research will need to examine whether energy labelling has a similar influence on the consumer behaviour of more extreme levels of socioeconomic deprivation (e.g. individuals living below the poverty line and/or experiencing food insecurity). Studies tended to examine impacts of energy labelling over short time periods and therefore longer-term effects will be important to consider. These points are of particular relevance, as a single study examined energy content of purchases in a US restaurant franchise up to 1 year after implementation of energy labelling [31]. There was an increasing trend in energy per transaction over the year follow up and there was a directional finding for this trend to be larger for restaurants in lower income census tracts. Further research examining whether the long-term consequences of energy labelling on consumer behaviour in real-world settings differs based on SEP is now recommended. In the present research we limited inclusion of studies to those that examined the effect of numeric energy labelling information only and found that the impact of labelling on consumer behaviour did not differ by SEP. It may be the case that when energy information is presented alongside other nutrition information and indicators rather than in isolation (e.g. traffic light labelling) these findings do not generalise and therefore understanding SEP differences in response to other types of food labelling may be informative.

## Conclusions

Overall, there was no convincing evidence that the effect energy labelling has on consumer behaviour significantly differs based on SEP. Further research examining multiple indicators of SEP and quantifying the long-term effects of energy labelling on consumer behaviour in real-world settings is now required.

## Supplementary Information

**Additional file 1. **Online supplementary materials.

## Data Availability

The study dataset and registered protocol is available on the Open Science Framework repository at https://doi.org/10.17605/OSF.IO/W7RDB

## Abbreviations

BMI: Body mass index
OOHF: Out of home food
SEP: Socioeconomic position
SMD: Standardised mean difference
