# Supplementary material for: Socioeconomic position and the effect of energy labelling on consumer behaviour: a systematic review and meta-analysis
Source: Int J Behav Nutr Phys Act. 2023 Feb 6;20:10. doi: 10.1186/s12966-023-01418-0 (PMC9903416; doi:10.1186/s12966-023-01418-0)
Supplement: Supplementary file 1 — Additional file 1. Online supplementary materials. [file 12966_2023_1418_MOESM1_ESM.docx]

Online supplementary materials

PubMed search terms used

((Energy OR kcal* OR calorie* OR kilojoule*) AND (label*) AND (Socioeco* OR educ* OR income OR deprivation OR poverty OR inequality)

To minimize irrelevant articles, in PubMed we will apply the species filters for ‘Human’ research only

Risk of Bias Assessment

Risk of bias will be assessed using a checklist adapted from generic study quality assessment tools, as items on any one existing risk of bias tool (e.g. Cochrane, ROBINS-I, Newcastle-Ottawa) were not comprehensive enough to cover all important methodological considerations for the types of studies included in the present review.

*Experimental studies*

a) Was random allocation to intervention vs. comparator conditions reported?

b) Did the study have a very small sample size? (n<20 in any SES group for the experimental or comparator condition in parallel arm studies)?

c) Were demand characteristics addressed in the study? (e.g. cover story used to ensure participants were unaware that the aim was to examine the effect of energy labelling, measurement of % of participants aware of study aims and majority not identifying aims, analyses conducted that examine whether results differ among participants aware of study aims vs. unaware participants)

d) Were outcome assessors blinded/unaware of the intervention/comparator condition received by study participants?

e) Were outcome data self-reported (e.g. dietary recall) as opposed to objectively measured (e.g. laboratory measured food intake, use of till receipts to infer sales)?

f) Was there evidence of potentially problematic levels of missing data? Availability of data from 90% (i.e. ≤10% missing) participants tends be sufficient. In instances of >10% missing data, if proportion of missing data is similar across conditions, then this would tend to be less problematic.

g) Were higher vs. lower SES groups different in terms of other potentially confounding demographics (e.g. BMI, ethnicity, gender, age)?

*Pre-post studies*

a) Did the study have a very small sample size? (n<20 per SES experimental vs. comparator condition)?

b) Were participants aware they were in a study prior to assessment of outcome?

c) Were outcome assessors (in instances where outcomes were not automated and instead dependent on interaction with participants or subjective rating by the researcher) aware of

the intervention/comparator condition received by study participants?

d) Were outcome data self-reported (e.g. dietary recall) as opposed to objectively measured (e.g. use of till receipts to infer sales)?

e) Was there evidence of potentially problematic levels of missing data? Availability of data from 90% (i.e. ≤10% missing) participants tends be sufficient. In instances of >10% missing data, if proportion of missing data is similar across conditions, then this would tend to not be problematic.

f) Were higher vs. lower SES groups different in terms of other potentially confounding demographics (e.g. BMI, ethnicity, gender, age)?

g) Were pre and post implementation comparison samples of participants different in terms of potentially confounding demographics (e.g. BMI, ethnicity, gender, age) If so, did analyses fail to account for these differences (e.g. use propensity score weighting)?

h) Were the same outlets sampled both pre and post energy labelling implementation?

i) Were there any additional changes to the study setting that coincided with the implementation of energy labelling (e.g. introduction of price promotions or menu changes)?

Additional Analyses

*Kcal selected*

When kcals selected was the outcome, as opposed to SMD (n = 16), kcal labelling led to a significant reduction of 39.79 kcal ([95% CI: 4.87 to 74.72], Z = 2.23, p = .025). The moderation by SEP was non-significant (X^2^(1) = 0.082, p = .774). In higher SEP this was 43.92 kcal [95% CI: -20.90 to 108.75] and in lower SEP this was 42.57 kcal [95% CI: -1.64 to 86.77].

*Risk of bias analyses*

One study was identified has having a higher risk of bias (based on bias for ≥75% of the individual bias indicators). When removing the effect sizes from this study the overall effect of kcal labelling (n = 24) was SMD = 0.080 ([95% CI: -0.025 to 0.185], Z = 1.49, p = .137, I^2^ = 74.6%). The moderation by SEP level was not significant (X^2^(1) = 0.317, p = .573). In higher SEP the effect was SMD = 0.049 [95% CI: -0.121 to 0.218], and in lower SEP the effect was SMD = 0.106 [95% CI: -0.021 to 0.233].

Examining effect sizes from studies which were randomised and assessed demand characteristics (n = 12) demonstrated a non-significant effect of kcal labelling (SMD = -0.038 [95% CI: -0.198 to 0.122], Z = 0.464, p = .643, I^2^ = 75.2%). The moderation by SEP level was not significant (X2(1) = 0.34, p = .558). In higher SEP the effect was SMD -0.087 [95% CI: -0.345 to 0.171] and in lower SEP the effect was SMD = 0.007 [95% CI: -0.196 to 0.210].

| Online Supplementary Table 1. Risk of Bias Ratings for Experimental Studies | | | | | | | | |
| --- | --- | --- | --- | --- | --- | --- | --- | --- |
| **Study Number** | **Study Reference** | **Random Allocation** | **Small sample size (N<20)** | **Demand characteristics addressed** | **Assessors blinded** | **Data self-reported** | **High levels of missing data** | **SES groups different in demographics** |
| **1** | Carbonneau et al., 2015 | Y | Y | Y | N | N | N | N |
| **3** | Dodds, 2014 | Y | N | N | Y | N | N | N |
| **4** | Antonelli & Viera, 2015 | Y | N | N | N | N | N | N |
| **5** | Marty, 2020 | Y | N | Y | Y | N | N | N |
| **6** | Marty, 2021a | Y | N | Y | Y | N | N | N |
| **7** | Marty, 2021b | Y | N | Y | N | N | N | Y |
| **8** | Maynard et al, 2017 | Y | Y | Y | Y | N | N | N |
| **9** | Al-Otaibi et al., 2021 | N | N | N | N | N | N | N |
| **10** | Morley et al., 2013 | Y | N | N | N | N | N | N |
| **11** | Walker et al., 2019 | Y | N | N | N | N | N | N |
| **15** | Masic et al., 2017 | Y | N | N | Y | N | N | N |
| **16** | Van Epps et al., 2021 | Y | N | N | Y | N | N | N |
| **17** | Robertson & Lunn, 2020 | Y | Y | Y | Y | N | N | N |

| Online Supplementary Table 2. Risk of Bias Ratings for Pre/post studies | | | | | | | | | |
| --- | --- | --- | --- | --- | --- | --- | --- | --- | --- |
| **Study Number** | **Study Reference** | **Small sample size (N<20)** | **Demand characteristics addressed** | **Assessors aware of intervention condition** | **Data self-reported** | **High levels of missing data** | **Pre/post samples demographics**  **same** | **Same outlets assessed pre/post** | **Additional changes pre/post** |
| **2** | Krieger et al, 2013 | N | N | N | N | N | N | Y | Y |
| **12** | Dumanovsky, 2011 | N | N | N | N | N | Y | Y | Y |
| **13** | Elbel, 2013 | N | N | N | N | N | Y | N | Y |
| **14** | Petimar et al., 2019 | N | Y | N | N | N | N/A | Y | Y |
